# Capturing domain knowledge from multiple sources: the rare bone disorders use case

**DOI:** 10.1186/s13326-015-0008-2

**Published:** 2015-04-17

**Authors:** Tudor Groza, Tania Tudorache, Peter N Robinson, Andreas Zankl

**Affiliations:** School of ITEE, The University of Queensland, St Lucia, Australia; Stanford Center for Biomedical Informatics Research, Stanford University, Stanford, USA; Institut für Medizinische Genetik und Humangenetik, Charité - Universitätsmedizin Berlin, Berlin, Germany; Children’s Hospital, Westmead, The University of Sydney, Sydney, New South Wales Australia

## Abstract

**Background:**

Lately, ontologies have become a fundamental building block in the process of formalising and storing complex biomedical information. The community-driven ontology curation process, however, ignores the possibility of multiple communities building, in parallel, conceptualisations of the same domain, and thus providing slightly different perspectives on the same knowledge. The individual nature of this effort leads to the need of a mechanism to enable us to create an overarching and comprehensive overview of the different perspectives on the domain knowledge.

**Results:**

We introduce an approach that enables the loose integration of knowledge emerging from diverse sources under a single coherent interoperable resource. To accurately track the original knowledge statements, we record the provenance at very granular levels. We exemplify the approach in the rare bone disorders domain by proposing the Rare Bone Disorders Ontology (RBDO). Using RBDO, researchers are able to answer queries, such as: “*What phenotypes describe a particular disorder and are common to all sources?*” or to understand similarities between disorders based on divergent groupings (classifications) provided by the underlying sources.

**Availability:**

RBDO is available at http://purl.org/skeletome/rbdo. In order to support lightweight query and integration, the knowledge captured by RBDO has also been made available as a SPARQL Endpoint at http://bio-lark.org/se_skeldys.html.

## Multiple perspectives over the same domain

Ontologies represent a formalised description of the concepts and relationships in a domain. For example, they can be used to model and capture knowledge around a particular set of hereditary disorders (e.g., bone dysplasias), in addition to their underlying genetic mechanisms (relating disorders to genes) and their observable traits (relating disorders to phenotypes). Over the course of the past decade, they have become one of the main mechanisms used in building intelligent systems and algorithms to support, among others, the study of cross-species phenotype networks [1-3], gene screening, prediction and prioritization [1,4] or disorder prediction [5,6]. This rising adoption, in particular by the biomedical community, has led to the proliferation of the number of ontologies published openly via repositories such as the NCBO BioPortal [7] or the Open Biological and Biomedical Ontology (OBO) Foundry [8]. An effect of this uptake has been the need for a community-driven process [9], which requires a shared understanding of the rules and primitives that govern the domain under scrutiny. More concretely, experts required appropriate mechanisms to update and evolve ontologies and the domain knowledge, in order to ensure an accurate knowledge transfer. A relevant example of such collaborative knowledge curation is the development of the International Classification of Disorders (ICD-11) [10], or efforts like the Gene Ontology [11], the Human Phenotype Ontology [12] or the International Classification for Nursing Practice (ICNP) [13].

Recognising the need for community-oriented knowledge curation does not, however, take into account that multiple communities may target, in parallel, the conceptualisation of the same domain. This, in turn, leads to ontologies that provide slightly different perspectives on the same knowledge. These perspectives differ in:

1. focus – the particular aspects of the domain – e.g., given a particular set of disorders, a community may focus more on the genetic mechanisms, while another one on the clinical presentation,
2. granularity – the level of detail considered in the knowledge modelling process – e.g., given the same context, the knowledge captured by a community may include the prevalence of the disorders or the age of onset, while in other cases this knowledge may be omitted, or
3. in the underlying interests or priorities of those creating them – for example, a community may just be interested in the clinical presentation of the disorders because of its focus on clinical diagnosis.

We are currently missing a key mechanism to allow us to create a comprehensive overview of the different perspectives on the domain knowledge.

Ontology integration is a field that has been extensively studied in the past. Although several definitions of “ontology integration” exist in the literature [14], it is usually referred (as is in our case) to the process of combining two or more ontologies about the same subject into a single unified ontology. One step in the integration process is to find the correspondences (a.k.a., mappings) between the semantically-related entities, which can be done manually or (semi-automatically). The ontology-matching topic that has seen an enormous amount of research, as shown by several comprehensive and thorough surveys that are available in the literature [15-18]. All these approaches are focused on trying to identify in a (semi-)automatic manner the correspondences between entities in different ontologies. This is not the focus of our work, as the mappings between the two resources were identified in a manual fashion by a domain expert. Even more, existing work on ontology integration does not keep track of the provenance of the integrated entities. This novel aspect of our work brings several benefits, which are discussed later in the paper.

In the biomedical domain, many ontologies create cross-references to other vocabularies, as a means to keeping track of both the mapping between terms, and, sometimes, provenance of a term. The UMLS Metathesaurus [19] provides the Concept Unique Identifiers (CUI) that represent the unique meaning of a concept and is used to map between terms from different source vocabularies. The Disease Ontology (DO) [20] reuses terms from different biomedical ontologies (MeSH, ICD, OMIM and NCI Thesaurus), either by inclusion or cross-mapping, and keeps track of their provenance by recording their source id as a property associated to the class. As such, the Disease Ontology provides a rich resource for semantically connecting phenotypic, gene and genetic information related to human disease. Even though, the Disease Ontology approach is somewhat related to ours, there are two significant differences: (i) The domains covered by the DO and our RBDO are different (general disease vs. rare bone disorders); (ii) Our focus is on storing the provenance at very granular level, including at the relationship level (as opposed to only at entity level), in a manner that is computable.

In this paper we present a model, and its associated implementation, aimed at integrating classifications of disorders developed by multiple entities, with a focus on the rare bone disorder domain. More concretely, we showcase the integration of the ORPHANET rare bone disorders classification [21] with the International Skeletal Dysplasia Society Nosology [22]. The resulting ontology – the Rare Bone Disorders Ontology – structures knowledge via the Simple Knowledge Organisation System (SKOS) vocabulary [23] and documents provenance via the Provenance Ontology [24]. From a conceptual perspective, the atomic elements of the classification integration resemble nano-publications [25], while RBDO acts as a coherent aggregation of such nano-publications. Finally, we describe a simple SPARQL query interface that enables the interaction with the knowledge, directly via SPARQL queries or via pre-built query templates.

## Current status in the rare bone disorders domain

### Rare bone diseases knowledge resources

The rare bone disorders domain is an example where the above-mentioned integration challenge can be observed and where developing a consolidated solution would benefit the all communities working on the topic. In this section, we discuss the existing domain knowledge representations together with their advantages and shortcomings.

The **International Skeletal Dysplasia Society** (ISDS) publishes every 4 years the Nosology of bone dysplasias [22] – based on the recommendation provided by a panel of experts. The Nosology classifies each disorder into a single group, based either on its clinical radiographic appearance or on its underlying molecular genetic mechanism. In some cases, this classification scheme proves to be too rigid, since disorders may easily be associated with multiple groups – due also to the lack of a complete overview of the knowledge surrounding them. Furthermore, the actual Nosology incorporates only the genotype of the domain, with no relationships to the characteristic phenotypes (which are implicitly present in the taxonomy).

The **Bone Dysplasia Ontology** (BDO) [26] aims to address some of the shortcomings of the Nosology by transforming it into an appropriate knowledge representation formalism. BDO maintains the taxonomical organisation of the Nosology and the genotype description but also includes disorder – phenotype associations compiled from the clinical synopses published in the Online Mendelian Inheritance in Man database (OMIM) [27]. More concretely, the authors annotate the clinical synopses with Human Phenotype Ontology (HPO) [12] concepts and link the resulting annotations to skeletal dysplasias in BDO. Overall, the Bone Dysplasia ontology acts as a consolidating agent between the ISDS Nosology and the OMIM phenotype description of the disorders.

The **Orphanet Consortium**, on the other hand, has also developed over the years a taxonomy of rare bone disorders that lists skeletal dysplasias as central elements. This taxonomy, more elaborated than the ISDS Nosology, is enriched with manually curated genotype and phenotype knowledge, the latter including an additional level of detail and mappings to HPO concepts (i.e., the degree of occurrence of the phenotype in the context of the disorder - e.g., Achondroplasia is associated “*very frequently*” to Macrocephaly).

### Differences in the representation of the current resources

There are several major differences between the BDO/ISDS and Orphanet that are worth noting. As a side note, we need to remark that the Orphanet classification is by default more generic and includes disorders that are not bone dysplasias. Our analysis, however, focuses strictly on those components of Orphanet that are common to both classifications. **1. Orphanet introduces a more fine-grained phenotypic annotation by specifying the frequency of occurrence of a phenotype in the context of a given disorder.**

**Orphanet:**



**BDO/ISDS:**

**2. The Orphanet taxonomy is more complex, nesting disorders up to 7 levels deep, as opposed to the 3 levels defined by BDO/ISDS.**

**Orphanet:**



**BDO/ISDS:**

**3. The disorder classification mechanism also differs.** Orphanet uses the hierarchy to denote both grouping of disorders, e.g., several disorders are part of the FGFR3 Chondrodysplasia Group, as well as sub-typing, e.g., Mesomelic dysplasia, Korean type is (conceptually) a type of Mesomelic dysplasia. Such groupings may occur as siblings in the hierarchy.

**Orphanet:**



**BDO/ISDS:**



**4. The number of bone dysplasias and their phenotype associations covered by the two sources differs significantly:** (i) there are 202 bone dysplasias present in BDO but not in Orphanet (i.e., 38.5% of the total of 524 disorders listed in BDO); (ii) BDO reuses 2,183 HPO concepts to describe phenotypic characteristics, while Orphanet reuses only 1,133 HPO concepts. **5. The actual disorder – phenotype associations are different between BDO and Orphanet.** For example, Adams-Oliver syndrome has 40 HPO concepts associated in BDO and 66 in Orphanet, only 14 of which are in common.

Both classification systems have been developed by domain experts and the apparent differences might reflect true differences in opinion between the expert groups. We do not suggest that this divergence is due to errors and that a ground truth should exist, as there is no easy way to reconcile them to arrive at such a ground truth. We do, however, advocate for building a model that is able to represent both classifications by recording the appropriate provenance and that enables researchers to use both sources in an integrated or comparative manner.

In the remainder of the paper we discuss the benefits of integrating diverse domain knowledge views and then propose a new ontology, named the Rare Bone Disorders Ontology (RBDO), which shows how to achieve this integration from a pragmatic perspective. The goal of RBDO is to provide a comprehensive overview of the rare bone disorders domain and to enable researchers and clinicians to analyse in a comparative manner the different perspectives on their genetic and phenotypic descriptions. RBDO is at the border between a domain ontology and an application ontology. On one hand, it can be used for reasoning tasks, similar to a domain ontology. On the other hand, it can also be used to serve specific integration purposes, subject to the underlying application. The current state of RBDO integrates BDO/ISDS 2010 (the latest to date) and Orphanet data May 2014.

Using RBDO, researchers will be able to answer queries, such as: “*What phenotypes describe a particular disorder and are common to all sources?*” or to understand similarities between disorders based on divergent groupings (classifications) provided by the underlying sources. As a remark, ’all sources’ refers in this context to the sources used for integration by RBDO. From a computational perspective, RBDO supports, among other things, the creation of personalised views over the domain knowledge (e.g., by choosing a particular source or combining several sources), which can subsequently be used for automated consistency checking and reasoning (e.g., classifying disorders based on their phenotype description). Conceptually, the model used by RBDO can be applied in any biomedical domain that may take advantage from the integration of various sources of knowledge, however, our current focus is only on bone disorders.

## Benefits of loosely integrated domain knowledge views

The previous section has noted various differences between the BDO/ISDS and Orphanet, including: (i) different taxonomical groupings; (ii) different ways of sub-typing disorders; or (iii) different phenotypic descriptions.

Here we discuss the benefits of using a light and flexible taxonomic representation to incorporate the different views on the domain knowledge, by maintaining both the granularity, as well as provenance.

- **Aggregated analysis:** Traditionally, ontologies externalise knowledge statements defined by an underlying community. On the other hand, an ontology that integrates the viewpoints of multiple communities creating and publishing knowledge in the same domain enables an aggregated analysis over this domain knowledge. For example, a researcher is able to understand and analyse the differences in phenotype descriptions between BDO/ISDS and Orphanet (in agreement or disagreement) or complement missing descriptions in one source with those defined by the other. Similarly, automated classification or reasoning mechanisms can take advantage of the multiple viewpoints definition of the knowledge and give a higher weight or impact to those statements that have been defined by both (or several) sources as opposed to those defined by a single source.
- **Extensibility:** Incorporating additional sources in a model that targets integration implies the analysis of the common and distinctive knowledge statements. This is then followed by an enrichment of the ontology with those statements that have not been defined yet, or by adding the new source as provenance to already existing statements. As an example, we intend in the near future to incorporate in RBDO also the classification and description of bone dysplasias according to Spranger et al. [28]. This differs, again, in various aspects, including the grouping strategy or association with different phenotypes and thus will provide yet another perspective on the bone dysplasia knowledge.
- **Backwards compatibility:** By keeping track of the provenance of all elementary knowledge statements, one is able to re-create the original model – as initially developed by a group / organisation – simply by building a view over the ontology from the perspective of the corresponding entity. For example, the original BDO/ISDS classification can be re-created by retaining from the current Rare Bone Disorder Ontology only those concepts and relations that were attributed to ISDS and OMIM. Such an operation may be desirable if classification according to a particular group / organisation is sought.

## Integrating multiple classifications and recording the associated provenance

A typical ontology consists of a hierarchy and a set of additional relations between its concepts. The hierarchy denotes, for example, the taxonomy of disorders, e.g., Acrodysostosis is a *sub-class* of Acromelic Dysplasias. On the other hand, the additional relationships may be used to related these concepts to other concepts introduced by the ontology, or to concepts defined by other ontologies. For example, in the Bone Dysplasia Ontology (BDO) we find Achondroplasia to be *characterised_by*Macrocephaly (HP:0000256) and to have *associated_gene*FGFR3.

The conceptual model we propose follows to a large extent the same direction, but introduces two modelling distinctions. First, we propose the use of the Simple Knowledge Organisation System (SKOS) [23] to capture the hierarchy of disorders published by different entities (e.g., BDO/ISDS and Orphanet) SKOS is a W3C recommendation and has been widely used for building lightweight classifications. It provides an alternative mechanism to rigid logical classifications and it enables a straightforward publishing of the same knowledge as Linked Data. Finally, it is conceptually easy to understand also by non-experts. Second, we impose the documentation of the provenance of all concepts and relationships in the ontology – in order to keep track of the originating source. We shall discuss and exemplify these two aspects in the remainder of this section.

### The knowledge engineering process

In order to build RBDO, we first had to gain a deep understanding of the similarities and differences between BDO/ISDS and Orphanet. Consequently, with the help of a bone dysplasia expert, we performed a manual comparative analysis of the two sources, which included: 1. a manual alignment of the disorders and groups of disorders (using a spreadsheet); 2. a manual inspection, followed by an intersection of the genotype knowledge associated with each disorder; and 3. a repetition of the previous step for phenotype knowledge.

**Figure 1:**
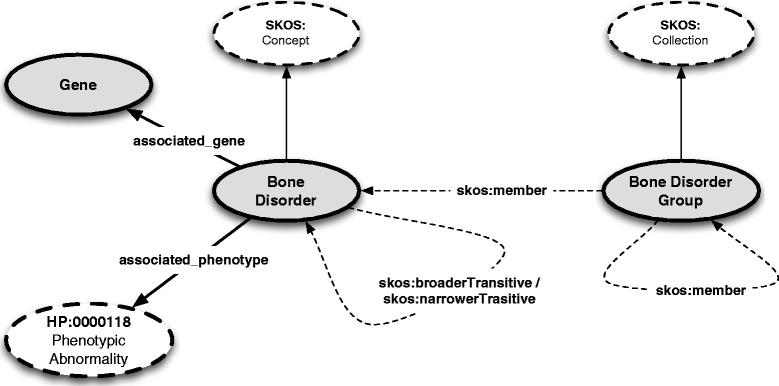
The upper-level structure of the Rare Bone Disorder Ontology.

Figure 1 depicts the major RBDO concepts and their associated relationships. These concepts have been adapted from the generic concepts initially defined in the Bone Dysplasia Ontology – i.e., Bone Disorder Group, Bone Disorder and Gene – in addition to re-using the knowledge captured by the Human Phenotype Ontology via its top level concept HP:0000118 (*Phenotypic abnormality*). The relations between Bone Disorder and Gene and Bone Disorder and HP:0000118, respectively, are conceptually the same as in BDO, i.e., a Bone Disorder has *associated_gene* any Gene and Bone Disorder has *associated_phenotype* any HP:0000118. A major difference to BDO is, however, the clear semantic distinction between group membership and disorder sub-typing via the SKOS vocabulary - discussed in detail later in the paper. Finally, provenance is recorded using the Provenance Ontology (PROV-O) [24], a World Wide Web Consortium recommendation, at all levels, for example, when defining bone disorders or associating a bone disorder with a gene.

It is important to note that our effort does not propose a novel ontology mapping approach. The literature contains a wealth of such solutions and we would refer the reader to [29] for a comprehensive overview. We, instead, focus on devising a conceptual mapping model for domain-specific taxonomies, with the added value of provenance, while the actual mapping process has been performed manually by a domain expert. This model is complementary to the existing automatic mapping approaches and provides an upper level scheme that can be used to integrate the mapping results.

### Modelling the taxonomy of bone disorders

In a typical ontology, the classification of disorders is modelled using the class – sub class relationship. The semantics of the sub-class relationship is one of specialization - for example, Mesomelic dysplasia Korean type or Mesomelic dysplasia Savarirayan type are subtypes of Mesomelic dysplasia. And while most ontologies use it appropriately, some use it with a different semantics, i.e., as a *part-Of* relationship, to show the membership of a particular disorder to a group – e.g., Acrodysostosisis a *sub-class of* – i.e., *member of* – Acromelic Dysplasias. Consequently, the same relationship is used with two different connotations, which does not capture accurately the semantics of the domain knowledge.

A second aspect that requires consideration is the fact that the same disorder can be classified into multiple groups, which may occur either due to the ambiguity introduced by the way in which the groups were formed, or by taking into account the knowledge provided by multiple sources. As an example in the first category, the FGFR3 Chondrodysplasia Group, defined by BDO/ISDS, collates bone disorders caused by a mutation in FGFR3 – hence the grouping is performed on a genetic basis. The Lacrimo-Auriculo-Dento-Digital syndrome (LADD), a disorder known to be caused also by a mutation in FGFR3, has however been placed in the Polydactyly-Syndactyly-Triphalangism Group in BDO/ISDS because of its clinical characteristics. Similarly, an example in the second category is the Holt-Oram syndrome: in BDO/ISDS the disorder is part of the Limb hypoplasia - reduction defects Group, while in Orphanet it represents a sub-type of the Heart-hand syndrome, which is classified under Dysostosis with limb anomaly as a major feature.

An elegant solution to address both above mentioned aspects is adopting the Simple Knowledge Organization System (SKOS). As mentioned above, SKOS enables the realisation of lightweight classifications via concepts that are easily understandable also by non-experts. For the purpose of our solution, two SKOS entities are of interest: SKOS Collection and SKOS Concept.

SKOS Concepts are the fundamental units of the SKOS vocabulary and they denote units of thought, i.e., ideas, meanings, objects, events, which underpin most of existing knowledge organizations systems. In our case, Bone Disorders, Genes and Phenotypes act as fundamental units of the ontology and of the domain (as per Figure 1). SKOS Collections provide a natural method to define meaningful groupings of concepts, such as Bone Disorder Groups. The actual grouping is realised by connecting a Collection to a Concept via the *skos:member* relationship (see Figure 1). Hence, from a general perspective Bone Disorder Groups are SKOS Collections, Bone Disorders are SKOS Concepts and Bone Disorder Groups have multiple Bone Disorder members. Moreover, in order to build multi-level hierarchies, SKOS Collections can, subsequently, be members of other SKOS Collections, via the same *skos:member* relationship. This is, for example, not possible if instead of using SKOS Collections we would have used SKOS Concept Schemes.

The advantage of using this modelling approach is that it enables us to place bone disorders into multiple groups, as well as to assign a clear semantics to group membership, and to aggregating groups of bone disorders and bone disorders, respectively, into the same group. The example above has already exhibited the multi-group membership within a taxonomy or across taxonomies. An example of multi-group membership with a mixture of both groups and disorders is the case of the Orphanet Dysostosis group (Orphanet:364559). Dysostosis has as members Dysostosis with predominant craniofacial involvement (Orphanet:93453) and Patellar dysostosis (Orphanet:93455), which are groups on their own, but also Congenital pseudoarthrosis of clavicle (Orphanet:66630), which is a disorder.

**Figure 2:**
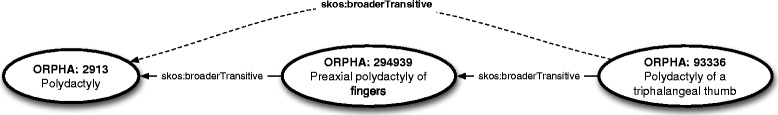
Example of disorder sub-typing via *skos:broaderTransitive* relations.Polydactly is broader than Preaxial polydactyly of fingers, which is broader than Polydactyly of a triphalangeal thumb. The use of *skos:broaderTransitive* in this triumvirate implies that Polydactyly is hence broader than Polydactyly of a triphalangeal thumb.

So far, we have addressed the issue of modelling groups of bone disorders. The second aspect relevant for the hierarchy is the disorder sub-typing, as per the previous example about Mesomelic dysplasia: *Korean type*, *Kantaputra type*, *Savarirayan type*. Similar to the grouping of disorders, SKOS provides a way to model this aspect using a clear semantics via the *skos:broaderTransitive* and *skos:narrowerTransitive* relationships – i.e., a Bone Disorder can be *skos:broaderTransitive* or *skos:narrowerTransitive* with respect to another Bone Disorder (see Figure 1). In practice, both relationships denote two elements: the broader / narrower association between Bone Disorders, e.g., Mesomelic dysplasia is broader conceptually than Mesomelic dysplasia, Korean type, as well as the transitivity aspect which ensures a natural hierarchy of disorders. An example of this order is presented in Figure 2: Polydactyly (Orphanet:2913) is broader than Preaxial polydactyly of fingers (Orphanet:294939), which in turn is broader than Polydactyly of a triphalangeal thumb (Orphanet:93336). Transitivity hence enforces Polydactyly (Orphanet:2913) to be, due to the existing broader relationships, also broader than Polydactyly of a triphalangeal thumb (Orphanet: 93336).

In addition to the hierarchical structure, the Rare Bone Disorders Ontology defines the relationships required to describe disorders by means of their underlying genetic and phenotypic characteristics. These relationships are *associated_gene* that connects a Bone Disorder to a Gene (e.g., Achondroplasia*associated_gene*FGFR3) and *associated_phenotype* that connects a Bone Disorder to Phenotypic abnormality (HP:0000118) – e.g., Achondroplasia*associated_phenotype*Macrocephaly (HP:0000256). Finally, additional relations are introduced to capture extra knowledge about Bone Disorders, such as the *mode_of_inheritance* relation, which relates a Bone Disorder to Mode of Inheritance (HP:0000005). All associations between Bone Disorders and their related concepts are modelled as OWL restrictions.

In conclusion, using the grouping and broader/narrower capabilities provided by SKOS, we are able to capture the entire knowledge encoded by both the ISDS Nosology (and hence BDO), as well as the Orphanet classification. A complete overview of the definitions and axioms defined by this new ontology are provided later in the paper.

### Recording provenance

Our approach records provenance at all granularity levels in the ontology – i.e., both when defining concepts, as well as when associating them via relationships. We believe that tracking provenance is currently a key aspect in domain-specific knowledge engineering and in particular in domains where the knowledge is not fully matured. Provenance offers a clear perspective on the origins of the diverse knowledge units, in addition to enabling a comparative view over those knowledge units that have been modelled by multiple sources. By embedding it within the definition of the ontological concepts, we can, by default, capture the entity that defines both the ontological concepts, as well as the diverse relationships that characterise them. We will discuss a concrete example further down in this section.

In order to capture provenance in RBOD, we adopted another well-known W3C recommendation – the Provenance Ontology (PROV-O). PROV-O provides the set of classes, properties and restrictions required to represent and share provenance. It consists of ten classes describing entities, agents and activities and a wide range of properties that cover aspects such as provenience, authorship, temporality, influence or roles – all in the context of provenance. For example, using PROV-O, one is able to specify the primary source of an information (*prov:hadPrimarySource*), to whom is the information attributed (*prov:wasAttributedTo*) or where was it derived from (*prov:wasDerivedFrom*).

**Figure 3:**
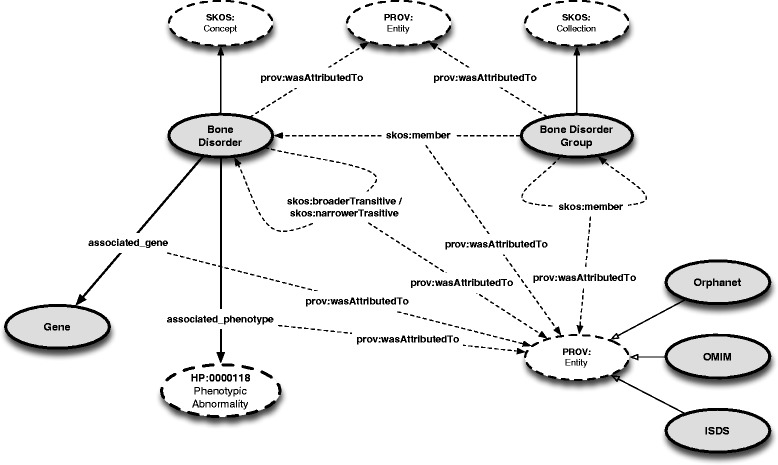
The upper-level structure of RBDO enriched with provenance information. As a note, the PROV: Entity concept is duplicated in the figure for readability purposes.
- a particular Bone Disorder as part of its classification – e.g., Achondroplasia is a Bone Disorder, Achondroplasia*prov:wasAttributedTo*ISDS, Orphanet
- group membership – e.g., FGFR3 Chondrodysplasia Group*skos:member*Achondroplasia; statement *prov:wasAttributedTo*ISDS
- broader / narrower relationships – e.g., Rubinstein-Taybi syndrome (Orphanet:783) *skos:narrowerTransitive*Rubinstein-Taybi syndrome due to 16p13.3 microdeletion (Orphanet:353281); statement *prov:wasAttributedTo*Orphanet
- the association between a Bone Disorder and a phenotype – e.g., Achondroplasia*associated_phenotype*Macrocephaly (HP:0000256); statement *prov:wasAttributedTo*ISDS
- the association between a Bone Disorder and a gene – e.g., Achondroplasia*associated_gene*FGFR3; statement prov:wasAttributedTo ISDS, Orphanet

Figure 3 depicts our ontology enriched with provenance. Using this model, one can add provenance to both entities defined in the ontology, as well as relationships created between these entities. More concretely, provenance is captured for:

- Bone Disorder Groups – e.g., FGFR3 Chondrodysplasia Group*prov:wasAttributedTo*ISDS

**Figure 4:**
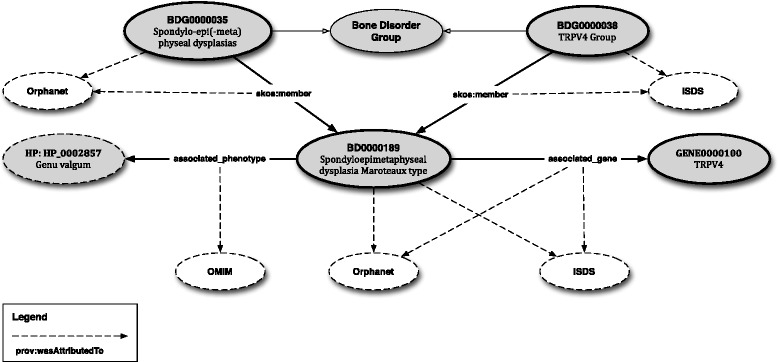
Excerpt from RBDO showing the placement, relationships and information attached to Spondyloepimetaphyseal dysplasia Maroteaux type. Dark ovals represent concepts in the ontology, and white ovals represent the original source of the concept or relationship.

All these aspects are depicted in an example in Figure 4, using Spondyloepimetaphyseal dysplasia Maroteaux type, its placement in BDO/ISDS and Orphanet and some of its associated knowledge.

## Implementation & availability

The Rare Bone Disorder Ontology (RBDO) aims to capture the domain knowledge in a computer-processable way and thus we opted for using a logical formalism to encode the relationships between the concepts. The resulting ontology class axioms not only encode the conceptual real-world knowledge (e.g., Achondoplasia has *associated_gene*FGFR3 and *associated_phenotype*Hydrocephalus (HP:0000238) or Lumbar hyperlordosis (HP:0002938)), but also enable users (and applications) to perform reasoning on patient instance data. The actual formalism we have used is the RL profile of the Web Ontology Language (OWL) 2 [30].

OWL 2 RL enables applications to perform scalable reasoning while at the same time maintaining most of the language’s expressiveness. The profile has been designed to cater for applications that allow a trade-off between the full expressivity of OWL 2 and efficient inference, since OWL 2 RL reasoning systems can be implemented using typical rule-based reasoning engines. Finally, this profile enables the use cases targeted by our ontology, i.e., consistency checking, class expression subsumption or conjunctive query answering, in a scalable and reliable way. We will use the Manchester syntax, a user-friendly syntax for OWL 2, to present the formal representation of the RBDO examples throughout this section.

Table 1 denotes the fact sheet of RBDO. The ontology defines two relationships and three own classes, the rest being re-used from Human Phenotype Ontology, SKOS and PROV-0. The two relationships (*associated_gene* and *associated_phenotype*, both defined using the Manchester syntax, in the listing below) enable us to relate bone disorders to genes and to phenotypes, respectively – the latter defined by HPO concepts.

**Table 1:** **The rare bone disorder ontology fact sheet**

Namespace	http://purl.org/skeletome/rbdo#
Prefix	RBDO
Scope	Bone disorders, genes, phenotypic characteristics in human
Format	OWL 2 RL
High-level classes defined	Bone Disorder, Gene, Bone Disorder Group
Relationships defined	associated_gene, associated_phenotype, mode_of_inheritance
Provenance entities defined	ISDS, OMIM, Orphanet
Number of bone disorders	1,146
Number of bone disorder groups	51
Number of genes	383
Total number of classes	1,586
Total number of phenotypes reused	2,750
Dependencies	HPO, SKOS, PROV-O
Annotations	rdfs:label, skos:altLabel; uniprot_id; omim_id; provo:wasAttributedTo
Availability	http://purl.org/skeletome/rbdo



The central concept of the ontology is the Bone Disorder class (see Figure 1 and listing below), which is defined as an entity characterised by a mode of inheritance (re-used from HPO) and by a series of associations with specific genes and phenotypes. Bone Disorders can be grouped into Bone Disorder Groups using the *skos:member* relation as shown in the previous section and can be associated with genes and phenotypes as per the above corresponding relation definitions. It is worth noting that the ontology defines Bone Disorders via a novel artefact introduced in OWL 2 – namely, punning – which allows an entity to be defined both as a class of concepts and as an instance of a particular concept at the same time. This was required in order to be able to accommodate the SKOS taxonomical structure, since a SKOS Concept is a class on its own, while bone disorders represent instances of SKOS Concepts. More details are shown in the example presented later in the section.



A key aspect of RBDO is the presence of provenance embedded within the logical definitions of the concepts. This is, in principle, possible due to another technical artefact introduced by OWL 2, which enables the definition of annotation assertions on class axioms and property restrictions. More concretely, as we will see in the next example, every class definition (and hence association with genes and phenotypes) or group membership definition is annotated with a provenance statement, which enables us, at any time, to track its original creator and eventually to create a specific provenance-driven view over the knowledge in the ontology.

In order to have a better understanding of the logical descriptions introduced in RBDO we detail below the example provided in Figure 4.



Firstly, let us note the definition of the Spondylo-epi(-meta)physeal dysplasias (BDG0000035) and TRPV4 Group (BDG0000038) bone disorder groups, as shown in the listings above. We can observe that the former has been defined by both ISDS and Orphanet, while the second is present only in ISDS. Furthermore, Spondyloepimetaphyseal dysplasia Maroteaux type (BD0000189) is listed as a member of both groups (see *skos:member*BD0000189 in the definition of each group). The *prov:wasAttributedTo* statements in the listings denote the use of annotation assertions to assign specific provenance to each group membership. The first statement captures the provenance of the group membership relation with Spondylo-epi(-meta)physeal dysplasias and assigns it to Orphanet, while the second has the same goal but in the context of the TRPV4 Group and ISDS.



The actual definition of the disorder is presented in the listing above. As a remark, for brevity, some of the axioms have been left out from this listing. Here, Spondyloepimetaphyseal dysplasia Maroteaux type is defined as a bone disorder belonging to both ISDS and Orphanet and which may be associated with Genu Valgum (HP:0002857) and with the TRPV4 gene (GENE0000100) – the definition of GENE0000100 is provided below.



Finally, the same mechanism for annotating the group membership provenance is used to annotate the class axioms and enforces the bone disorder to be defined in the context of the association with some phenotypes and genes. The *prov:wasAttributeTo* statements using in the listings above capture the provenance of the association with Genu Valgum (HP:0002857) and assigns it to OMIM, as well as the provenance of the underlying genetic mechanism – i.e., the association with the TRPV4 gene (GENE0000100) and assigns it to both ISDS and Orphanet.

## Publishing bone dysplasia Linked Data

As shown and discussed in the previous section, the Rare Bone Disorders Ontology has been made available in OWL 2 RL format in order to support a smooth transition to a specific entity-oriented view (i.e., BDO or Orphanet). This would then enable direct consistency checking and classification based on the current ontology axioms. The same knowledge, however, can also be published in a lighter format – a format focused on easy integration and query. Consequently, we have also transformed the ontology into a query and integration endpoint using a Linked Data approach.

The Linked Data initiative [31] has initially emerged from the need of making data trapped within legacy systems openly available on the Web for reuse and integration purposes. The proposed publishing process relies on four simple principles:

- Use unique identifiers (URIs) to denote entities/concepts/etc
- Use retrievable Web identifiers to enable both humans and machines to look up and access the entities corresponding to these identifiers
- Use structured formats (i.e., machine processable) to represent the data – or more precisely RDF – Resource Description Framework [CITE]
- Include links to other entities

The initiative has proved to be very successful, and has led to a massive growth in Linked Data datasets now available in RDF format on the Web – over 300 datasets of which around 20% are only in Life Sciences and include among others UniProt [32], NCBI Gene, KEGG Pathway, OMIM or Reactome [33]. The de facto language used to access and integrate this data is SPARQL (Simple Protocol and RDF Query Language) [34], which provides a set of specifications for querying their underlying graph structure.

The literature consists of several good examples of using integrated data to achieve particular goals in specific contexts. For example, Taboada et al. [35] have integrated SNOMED CT, OMIM and HPO and a patient clinical dataset in order to bridge the gap between domain specific phenotype descriptions and clinical data in the context of Cerebrotendinous Xanthomatosis. Consequently, they were able to successfully infer bidirectional phenotype-genotype relations in their domain of interest – e.g., “*What are the genetic variants that have been associated with a combination of traits?*”. Similarly Zhu et al. used Linked Data to perform drug data normalisation in the Pharmacogenomics Knowledge Base (PharmGKB) [36], while Kiefer et al. [37] mined genotype-phenotype associations in Gene Wiki Plus and OMIM, with a focus on commonly occurring chronic diseases.

**Figure 5:**
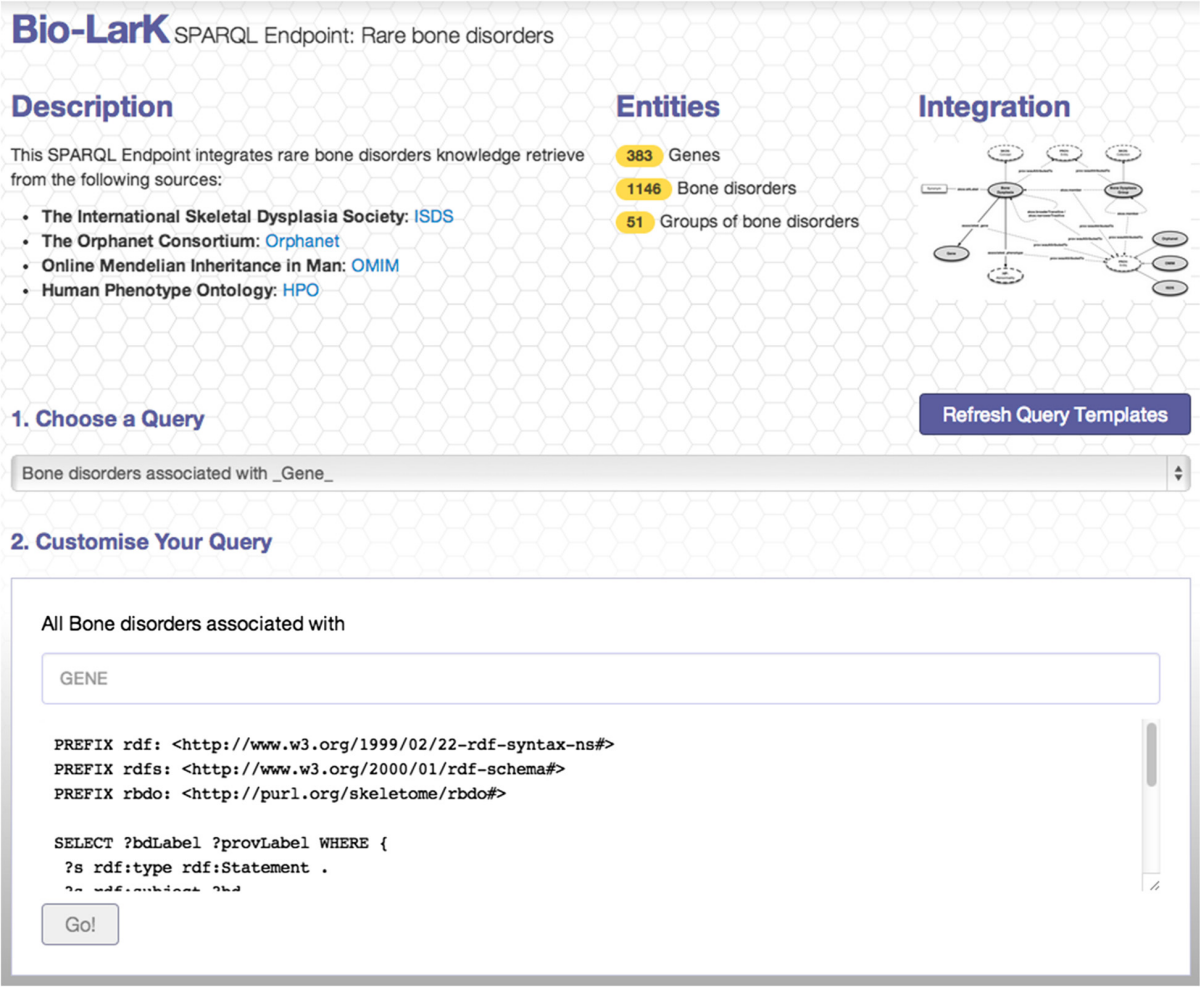
Main user interface of the Rare bone disorders SPARQL Endpoint. Users can select an existing query template to run, or write their own SPARQL queries.

**Figure 6:**
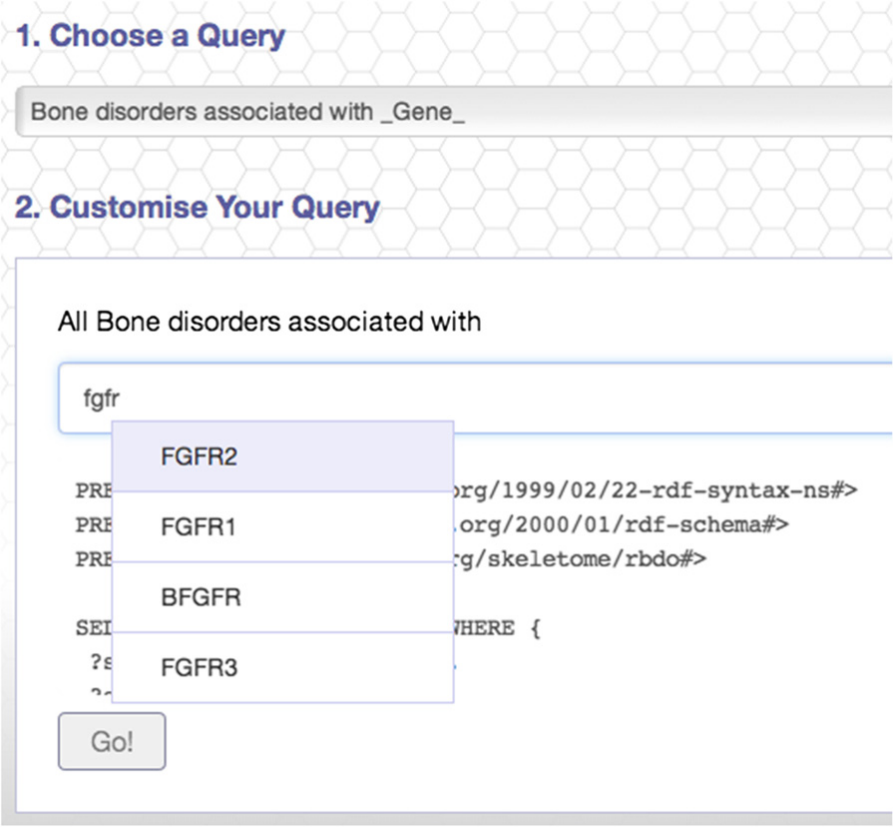
Example of query via autocompletion: “*All bone disorders associated with a chosen GENE*”.

**Figure 7:**
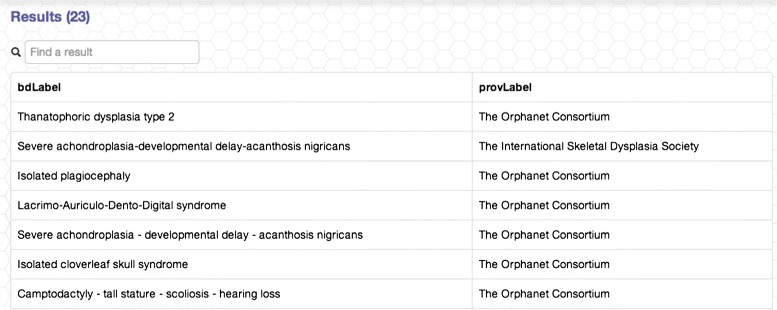
Example of results returned for the query “*All bone disorders associated with FGFR3*”.

In order to support this initiative and enable future integration of the bone disorders knowledge, we have published RBDO as a SPARQL Endpoint – available at: http://bio-lark.org/se_skeldys.html. Furthermore, we have also built a simple query interface to provide researchers and clinicians with the opportunity of exploring it – as shown in Figure 5. The interface uses a set of customisable templates that guide the user in building the desired queries. Currently, it offers a direct access to answering questions, such as “*Bone Disorders associated with a given GENE*” or “*Bone Disorders associated with a given PHENOTYPE*”. For example, given the first type of query, a researcher needs only to select from the existing gene list the desired one (as depicted in Figure 6). The interface then translates it into SPARQL and executes the query. The results are provided in a tabulated form (see Figure 7) and include the provenance of the disorder - gene association and a rapid filtering functionality, in the case in which a particular disorder is sought.

The Bone Disorders SPARQL Endpoint is the first initiative of this kind in the rare bone disorder domain and represents only the first step in our integration plan. Next steps include the integration of this knowledge with other datasets of direct interest, like KEGG Pathway (defining pathway – gene – disorder associations), Cell Cycle Ontology [38] (describing the cell cycle process inclusive of GO annotations) and Gene Ontology (defining among others biological and molecular functions). Consequently, the list of query templates will be enriched with new elements to enable an exploration of phenotype - pathway associations or disorder - phenotype - biological functions associations. Finally, to provide an even more comprehensive view over the domain, we intend to integrate bone dysplasia mouse genotype and phenotype knowledge from KEGG, Mouse Genome Informatics, the Mammalian Phenotype ontology [39] and the MP – HPO alignments, which will enable the unique opportunity of executing cross-species genotype and phenotype queries.

## Discussion

In this section, we discuss some of the challenges encountered in building RBDO, as well as some of its current limitations.

RBDO, as any other domain ontology, relies on the interpretation of the domain knowledge, interpretation which is then captured into the ontological definitions. In our case, this raised challenges when performing the manual mapping process, and especially when deciding the nature of some of the concepts in Orphanet – i.e., groups vs. non-groups. Here, the decision has been taken subject to the children of the concept under scrutiny. If the children represented particular types of the same disorder (see the Mesomelic dysplasia examples earlier in the paper), the concept has been defined as a Bone Disorder, and its children were sub-typed using *skos:broaderTransitive* relations. If, however, the children were a mixture of possible groups and disorders well-defined (using also BDO/ISDS as background knowledge), then the concept was defined as a Bone Disorder Group.

The goal of RBDO is to provide the scaffolding required to capture knowledge defined by several sources. Since this knowledge is not necessarily aligned (as in our use case), we may encounter logical conflicts when trying to consolidate it. For example, one source may define a bone disorder to be associated with a particular phenotype, which on its own, may be disjoint with another phenotype that is associated to the same disorder by another source. Currently, RBDO does not contain such conflicts because of the lack of a finer-grained definition of the disorder - phenotype associations and of the underlying phenotypic concepts. However, one of the major advantages of RBDO is that it is able to deal with this aspect, simply by allowing conflicting information to exist and recording its provenance. We believe that such information is valuable and should be maintained because it allows researchers to get a better understanding of the rationale behind the current classification of disorders. If logical consistency is sought, this can be achieved either by filtering the RBDO knowledge according to a single source, or by manually resolving existing conflicts when integrating multiple sources.

A final remark is worth mentioning about the sustainability and evolution of RBDO. The initial Bone Dysplasia Ontology has been published and used to generate the SKELETOME knowledge base – a community-driven platform for knowledge curation in the skeletal dysplasia domain. This platform implements an editorial process to describe and enrich knowledge around bone dysplasias and enables a continuous expert-based evolution of the underlying ontology. We plan in the near future to expand the SKELETOME knowledge based to incorporate RBDO, including the functionality required to add additional sources and to discuss conflicting knowledge. All interested researchers and clinicians will have access to SKELETOME and will be able to contribute to the curation and evolution of this rare bone disorders source of knowledge.

## Conclusions

In this paper, we have presented our effort in building and publishing an innovative model for capturing domain specific knowledge from multiple information sources using the rare bone disorders domain as our use case. We have shown how multiple, diverging taxonomies can be elegantly integrated using SKOS structures, as well as how to record provenance at all ontology definition levels – ranging from group membership, disorder sub-typing or disorder characterisation. The model has been implemented as a OWL2 RL ontology, to enable logical formalisation of the knowledge. Furthermore, the same knowledge has also been published in a lightweight format using Linked Data, to support immediate integration and querying.

A major limitation of our current work is the restricted range of queries that can be executed on the published SPARQL endpoint – in principle, this supports only basic interrogations on the association of bone disorders to phenotype and genotype. Our future efforts will, however, focus on continuously curating and expanding the knowledge in the rare bone disorders domain by integrating additional information sources including both human and cross-species data. This will enrich significantly the types of possible queries and will enable us to develop targeted knowledge discovery and exploration tools.
